# Iron Fertilization Can Enhance the Mass Production of Copepod, *Pseudodiaptomus annandalei*, for Fish Aquaculture

**DOI:** 10.3390/life13020529

**Published:** 2023-02-15

**Authors:** Guo-Kai Hong, Jimmy Kuo, Kwee Siong Tew

**Affiliations:** 1 Graduate Institute of Marine Biology, National Dong Hwa University, Pingtung 944401, Taiwan; 2 National Museum of Marine Biology & Aquarium, Pingtung 944401, Taiwan; 3 International Graduate Program of Marine Science and Technology, National Sun Yat-sen University, Kaohsiung 80424, Taiwan; 4 Department of Marine Biotechnology and Resources, National Sun Yat-sen University, Kaohsiung 80424, Taiwan; 5 Institute of Marine Ecology and Conservation, National Sun Yat-sen University, Kaohsiung 80424, Taiwan

**Keywords:** copepod, *Pseudodiaptomus annandalei*, inorganic fertilization method, iron, aquaculture, fish larval live feed

## Abstract

Copepods are proven nutritious food sources for the mariculture/larviculture industry, however, unreliable methods for mass production of copepods are a major bottleneck. In this study, we modified a previously reported inorganic fertilization method (N: 700 μg L^−1^ and P: 100 μg L^−1^) by the addition of iron (Fe: 10 μg L^−1^, using FeSO_4_·7H_2_O) (+Fe treatment) and compared its suitability for copepod culture (*Pseudodiaptomus annandalei*) to the original method (control). The experiment was conducted outdoors in 1000 L tanks for 15 days. The addition of iron prolonged the growth phase of the phytoplankton and resulted in the production of significantly more small phytoplankton (0.45–20 μm, average 2.01 ± 0.52 vs. 9.03 ± 4.17 µg L^−1^ in control and +Fe, respectively) and adult copepods (control: 195 ± 35, +Fe: 431 ± 109 ind L^−1^), whereas copepodid-stage was similar between treatments (control: 511 ± 107 vs. +Fe: 502 ± 68 ind L^−1^). Although adding iron increased the cost of production by 23% compared to the control, the estimated net profit was 97% greater. We concluded that inorganic fertilization, with the addition of iron (Fe: 10 μg L^−1^), could be an effective method for the mass production of copepods for larviculture.

## 1. Introduction

Larviculture is the first step in aquaculture. Over the decades, billions of food-fish larvae belonging to only a small number of species have been produced, while the larvae of the majority of other attempted fish species have failed to thrive [1]. Among the numerous difficulties faced in fish larviculture, the primary challenge is to provide live food of suitable size and nutritional quality to the first-feeding larvae [2,3,4,5]. Traditionally, rotifers, *Artemia* nauplii, and copepods have been provided as prey for fish larvae [1,6,7]. Although rotifers and *Artemia* are relatively easy to culture, their culture vessels must often be enriched with microalgae to increase their nutritional value, especially with respect to HUFA (highly unsaturated fatty acids), DHA (docosahexaenoic acid, 22:6n-3), and EPA (eicosapentaenoic acid, 20:5n-3), which are essential for the survival and growth of fish larvae [8,9]. Copepods, on the other hand, have a fatty acid composition already dominated by DHA, EPA, and saturated fatty acids [10,11], and many studies have confirmed their nutritional value for larval fish [9,12,13].

Copepods are one of the more common live feeds used in aquaculture [14,15], such as *Acartia tonsa* [16], and *Cyclopina kasignete* [17] species. The majority of commercial copepod products are collected from extensive outdoor culture ponds or as a by-product of fish ponds [18,19,20,21]. To enhance copepod production in outdoor culture ponds, various organic materials such as animal manure [22], soybean meal [23], alfalfa meal [24], and fish solubles [25] have been used traditionally to fertilize the ponds. Through the decomposition of the organic matter by bacteria, nutrients such as nitrate and phosphorus are released into the water, thereby enhancing phytoplankton growth, and the microalgae are then consumed by the copepods [22,26]. Production of this type is not reliable in terms of both copepod quantity and quality [18] since the nutrient contents and the lengths of decomposition of various organic fertilizers vary. Large-scale use of organic fertilizers leads to negative side effects such as the accumulation of nitrogenous wastes [27,28,29] causing the proliferation of harmful algae, eutrophication of water bodies [30,31], deteriorating water quality due to low oxygen [32], and high pH [25], and other extreme conditions in the ponds. In addition, different types of fish pathogens such as the microsporidian parasite *Enterocytozoon hepatopenaei* and the bacterium *Vibrio parahaemolyticus* can grow in such conditions [25], which might cause mass mortality of larval fishes when fed with those products. The commercial products grown in extensive outdoor culture ponds are not pure culture but are mixtures of different groups of crustaceans such as cyclopoids, calanoids, cladocerans, and ostracods [25]. These mixtures of zooplankton are not the ideal food for a lot of larval fish with very small mouth gapes [4,25,33]. Therefore, alternative applications of inorganic nutrients need to be explored in the mass production of copepods.

Inorganic fertilization has been widely used in agriculture on various crops but is relatively little used in aquaculture [32,33,34,35,36,37]. Previous studies have shown that compared to the traditional organic fertilization methods, the addition of inorganic nitrogen and phosphorus to aquaculture ponds results in fewer filamentous blue-green algae, but enhances more of the small unicellular algae that serve as the main food source for different sizes of zooplankton [4,35,36,37,38,39]. Using the inorganic fertilization method in culturing coral reef fish larvae has revealed that unicellular diatoms such as *Coscinodiscus* spp., *Navicula* spp., *Nitzschia seriata,* and *Nitzschia* spp. could be enhanced, and more small size zooplankton could be produced [4,33]. In order to mass produce unicellular phytoplankton for zooplankton, pure cultures of several algal species, such as *Isochrysis galbana*, *Nannochloropsis oculata*, and *Tetraselmis chui* have been developed [1]. The monoculture protocol of these algal species has been applied in many studies. Laboratory-scale attempts to augment the production of copepods using algal culture media such as F/2 medium [40] have been used in culturing *Bestiolina similis* [41], *Schmackeria poplesia* [42], and *A. grani* [43]. The results were encouraging, but using the algal culture media would not be cost-effective for industrial-scale mass rearing of copepods [44]. Moreover, monoculture of different algal species may occupy large space in an aquaculture facility, thus reducing the capacity to culture the intended copepod.

To date, only a few efforts at mass production of copepods using inorganic fertilization have been reported. Mischke and Zimba [26] found that applying only inorganic fertilizer to channel catfish nursery ponds at an initial concentration of ∼20 kg/ha N and 2 kg/ha P, followed by twice a week applications of half the initial quantity for 3–4 weeks, increased the concentration of zooplankton suitable as feed for fish with larger mouth gapes. Hong and Tew [25] evaluated the feasibility of using 700 µg L^−1^ N and 100 µg L^−1^ P for mass production of the calanoid copepod *Pseudodiaptomus annandalei* in 1000 L culture tanks, with the results that pathogen-free copepodites and adult copepods were significantly higher than with organic fertilization. Other benefits such as no development of filamentous algae in the tanks and lower total operating costs made it a profitable method for commercial copepod producers [25].

Iron (Fe) is involved in many physiological functions such as photosynthesis [45,46] and nitrogen absorption [47,48] and is a limiting nutrient for phytoplankton growth [49,50,51]. In addition to iron supplementation in aquaponic systems [52], dietary supplementation with inorganic iron has been tried in fish [53]. Adding iron has been shown to be effective in increasing the productivity of cultured phytoplankton [54], and also influences the fatty acid and sterol composition of phytoplankton [55]; this in turn affects the egg production rate of copepods that feed on the algae [55]. Until now, there has been no study on the effects of adding iron to aquaculture media. Since the inorganic fertilization method could potentially be very useful for the mass production of copepods [25], combining it with the addition of iron might further enhance copepod production.

The immediate goal of the present study was to assess how well the inorganic fertilization method we previously employed for the mass production of copepods [25] can be optimized by adding iron as culturing proceeds. The long-term goal is to improve the methodology for providing large quantities of larval and adult copepods for use in larviculture and thereby advance the large-scale rearing of additional fish species in aquaculture in the future.

## 2. Materials and Methods

### 2.1. Experimental Setup

We conducted the experiments outdoors at the National Museum of Marine Biology & Aquarium, Taiwan, in June, using ten 1000 L round fiberglass tanks that were filled with unfiltered natural seawater. After filling the tanks, no additional water changes or flows would be made for the entire experiment period. The inorganic nitrogen and phosphorus concentrations of each tank were measured daily and maintained at N: 700 μg L^−1^ and P: 100 μg L^−1^ for all tanks. Among them, five served as controls (Control, N = 5) while the iron was added to the other five at a concentration of 10 μg L^−1^ daily (+Fe treatment, N = 5). The sources of N and P were NH_4_NO_3_ and H_3_PO_4_ (SIGMA-ALDRICH, St. Louis, MO, USA) [25], respectively, whereas iron was added as FeSO_4_·7H_2_O (J.T. Baker, Radnor, PA, USA) [56]. Adult calanoid copepods, *P. annandalei*, which is commonly found in the coastal areas in Taiwan and is the dominant copepod species in the waters adjacent to the museum [25], were inoculated in each tank at a density of 10 ind L^−1^ on day 1. Previous studies have confirmed that the species is suitable prey for larval fishes [1,57,58,59] and pose no threat to their predator. The experiment lasted for 15 days since our objective is to mass produce the copepod in a short period of time and harvest them at the highest density possible (around day-15 from previous experiments) and restart the culturing process. We did not inoculate monoculture algae, therefore the algal compositions grown in the tanks were those from the natural seawater at the start of the experiment. 

### 2.2. Physicochemical Analyses

The temperature, pH, dissolved oxygen (DO), and salinity were measured daily at 1100~1300 h with a handheld multi-parameter meter (YSI Professional Plus, YSI, Yellow Springs, OH, USA) during the entire experiment, same procedures as in [25].

The dissolved inorganic nitrogen (NH_3_-N, NO_2_-N, and NO_3_-N), phosphorus (PO_4_-P), and total Fe concentrations of water samples that had been filtered through 0.45 μm filter paper were analyzed daily for each tank. We used HACH water analysis products (HACH, Loveland, CO, USA) such as the ammonia kit (salicylate method 8155), nitrite kit (diazotization method 8507), phosphorus kit (ascorbic acid method 8048), and total iron kit (FerroVer^®^ method 8008) in the analyses, and measured the nutrient concentrations with a spectrophotometer (Synergy H4 Hybrid Reader, BioTek Instruments, Winooski, VT, USA) [25]. Nitrate (NO_3_^−^) was reduced to NO_2_^−^ [60] before using the HACH nitrite kit.

### 2.3. Biological Analyses

Chl a-bearing algae were separated into two size categories: 0.45–20 μm and >20 μm, by filtering 200 mL of seawater taken daily from each tank through filter paper (Advantec, Tokyo, Japan) of two pore sizes, 0.45 μm, and 20 μm, respectively. A 0.45–20 μm fraction was calculated by subtracting the amount of Chl a on 20 μm filter paper from those on 0.45 μm filter paper. A previous study found that culture tanks could become overgrown with filamentous algae [25]; therefore, in the present experiment, we collected benthic algae from an area of 7.5 cm × 2.5 cm of the wall (30 cm below the surface) and bottom (150 cm below the surface) of each tank on day 15. The phytoplankton and benthic algae were then extracted with acetone [4,33], and the Chl a concentrations of the extracts were measured with a spectrophotometer (Hitachi U-5100, Hitachi, Tokyo, Japan). The taxonomical composition and density of the phytoplankton were counted and identified under a light microscope to the nearest taxon possible [4,33,35].

To assess the zooplankton, a 1 L water sample was taken daily from each tank after using a stirrer to vigorously disturb the tanks for 30 s. Samples were fixed using 5% formaldehyde, then the water was filtered through a 25 μm mesh, and all the zooplankton (non-copepod, copepod nauplii, and copepodids + adult copepods) were identified and counted under a compound microscope. We dried the copepods from each sample at 70 °C for 3 days in an oven to obtain the total copepod dry weight, and measured them using a 6-digit digital analytical microbalance (XP2U Ultra Micro Balance, Mettler-Toledo, Columbus, OH, USA), and used the formulae from Blanda, et al. [61] and Rayner, et al. [62] to calculate the total daily dried weights.

The population growth rates (r) for total copepod between control and +Fe treatments were calculated from the exponential phase of each population growth using the equation: r = *ln*(N_t+1_ − N_t_)/t, where N_t_ = population density at time t, and N_t+1_ = population density after time t+1, and t is the time interval (d) [63].

The cost per unit dry weight of copepods was calculated for each treatment by dividing the total cost of the fertilizers (N, P, and Fe) by the total dry weight of copepods produced during the experimental period. We also calculated the net income over the 15-day period, assuming the sale at the average market price (2.20 NT$ g^−1^) [25] of the total quantity of copepods produced per tank, minus the total cost per tank.

### 2.4. Statistical Analysis

We used one-way repeated measures analysis of variance (RM-ANOVA) to determine the effects of adding iron on various biological and physicochemical parameters, with sampling date treated as the repeated factor. Benthic algal Chl a concentrations, total cost, and net income of copepods were compared between treatments by using *t*-tests. In order to meet the assumptions of normality and homogeneity of variance, we transformed all the data when necessary. We used SigmaPlot 12.5 (SPSS 1997) in all statistical analyses, and α = 0.05 was considered statistically significant [25].

## 3. Results

**Figure 1:**
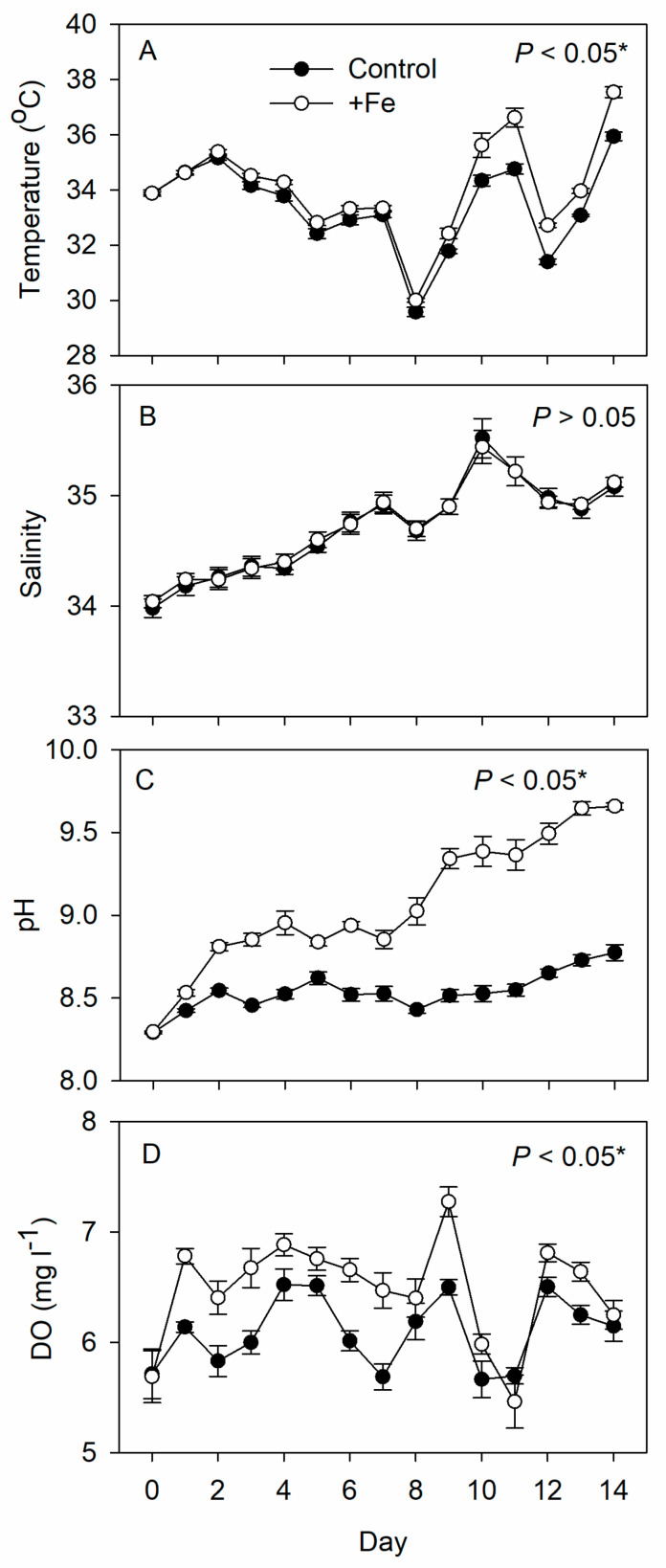
Daily measurements of (**A**) temperature, (**B**) salinity, (**C**) pH, and (**D**) dissolved oxygen in the control (N = 5) and +Fe (N = 5) treatment tanks during the 14-day experimental period (mean ± SD). The *p*-value in each panel indicates the significance level (*, α = 0.05) of the treatment effect based on repeated measures ANOVA.

During the experiment, the temperature, pH, and DO were significantly higher in the +Fe treatment tanks than in the control tanks, while the salinity was similar between the two (Figure 1).

**Figure 2:**
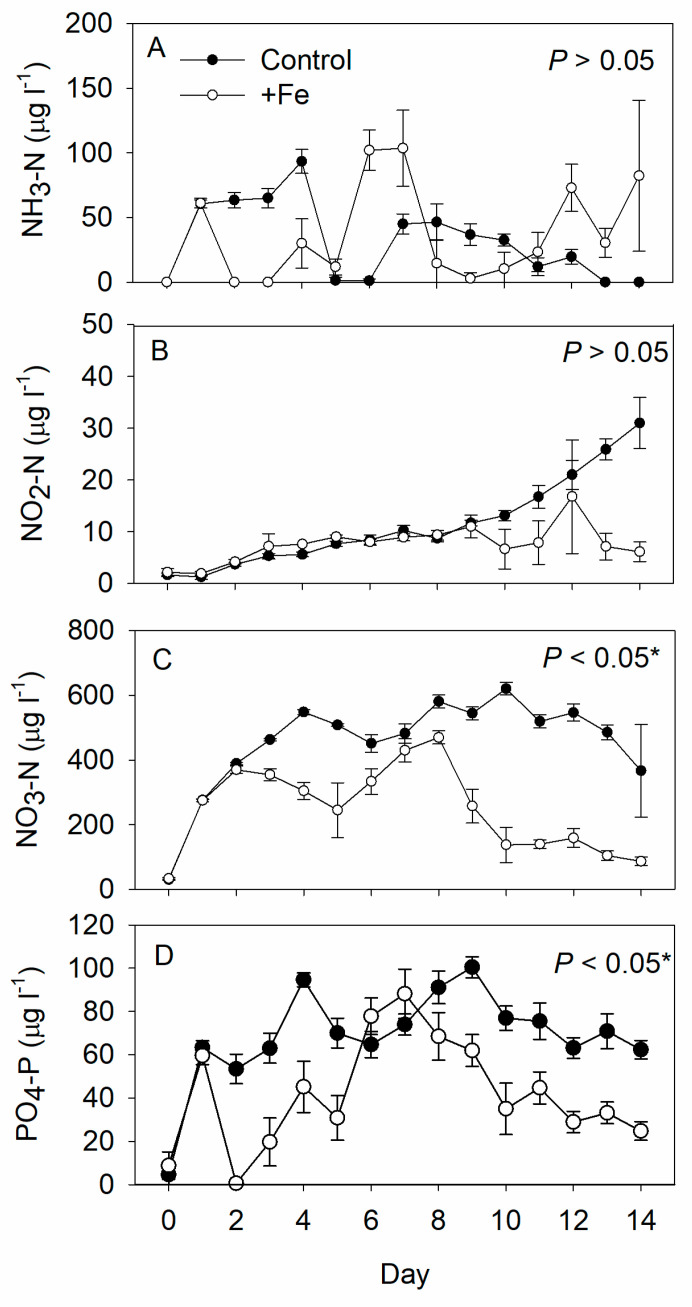
Daily measurements of (**A**) ammonium-nitrogen (NH_3_-N), (**B**) nitrite-nitrogen (NO_2_-N), (**C**) nitrate-nitrogen (NO_3_-N), and (**D**) phosphate (PO_4_-P) concentrations in the control (N = 5), and +Fe (N = 5) treatment tanks during the 14-day experimental period records (mean ± SD). The *p*-value in each panel indicates the significance level (*, α = 0.05) of the treatment effect based on repeated measures ANOVA.

The NO_3_-N and PO_4_-P concentrations were significantly lower in the +Fe treatment tanks than in the controls, while the NH_3_-N and NO_2_-N concentrations were similar between the two (Figure 2). Although 10 μg L^−1^ Fe was added daily to each +Fe treatment tank (i.e., 0.05 g FeSO_4_·7H_2_O per tank), the concentration of iron in these tanks always remained below the HACH iron kit’s detection limit.

**Figure 3:**
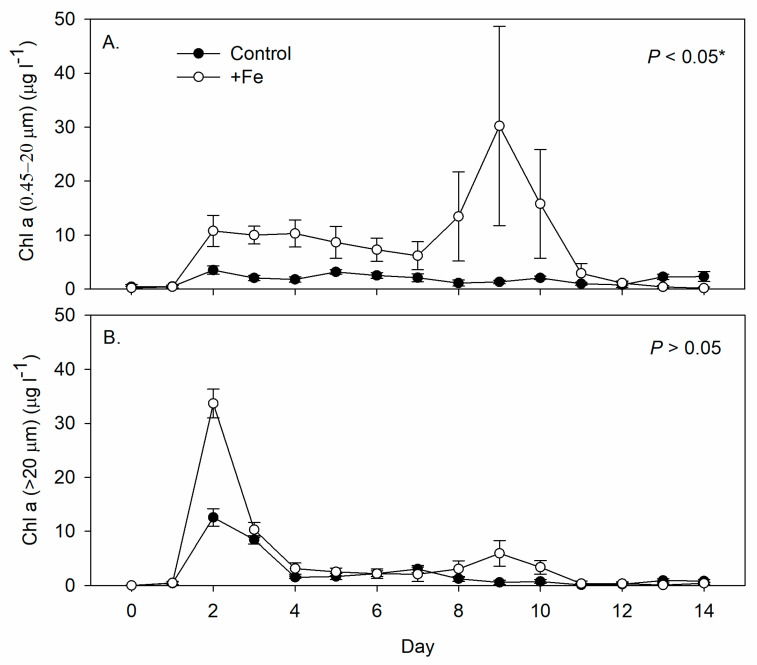
Daily measurements of (**A**) smaller phytoplankton chlorophyll a concentration (0.45–20 µm, µg L^−1^), and (**B**) larger phytoplankton chlorophyll a concentration (20 µm, µg L^−1^) in the control (N = 5) and +Fe (N = 5) treatment tanks during the 14-day experimental period (mean ± SD). The *p*-value in each panel indicates the significance level (*, α = 0.05) of the treatment effect based on repeated measures ANOVA.

The Chl a concentration of smaller phytoplankton (0.45–20 μm) was significantly higher in the +Fe treatment tanks than in the control tanks (Figure 3A), while that of the larger phytoplankton (>20 μm) was similar between the two (Figure 3B). Phytoplankton of both sizes began to grow on day 2 in both treatments (Figure 3). At the beginning of the experiment (Day 0~2), about 95% of the phytoplankton were diatom *Chaetoceros* spp. and shifted to green algae *Chlamydomonas* spp. and dinoflagellates *Gymnodinium* spp. in both treatments. *Chlamydomonas* spp. remained at about 74~78%, while *Gymnodinium* spp. at 18~24% between Day 4~12 in the control tanks; whereas in +Fe treatment tanks, *Chlamydomonas* spp. composed of more than 95% of the total phytoplankton densities from Day 8 until the end of the experiment. Although no visible benthic filamentous algae were found with either treatment, the benthic algae on the wall and bottom in the +Fe treatment tanks (2.83 ± 0.63 and 9.43 ± 1.27 µg cm^−2^, respectively) were significantly higher than in the control tanks (0.25 ± 0.15 and 3.88 ± 0.54 µg cm^−2^, respectively).

**Figure 4:**
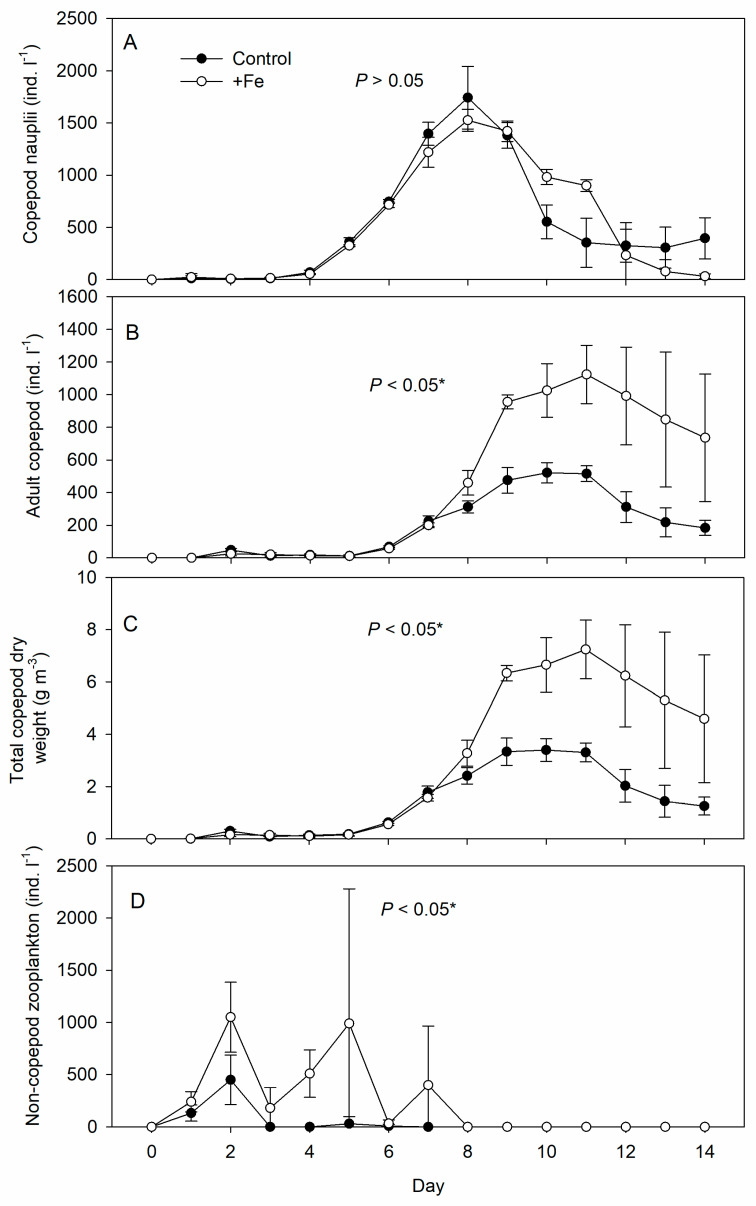
Daily measurements of abundance (density) of (**A**) copepod nauplii, (**B**) adult copepod, (**C**) total copepod dry weight, and (**D**) non-copepod zooplankton in the control (N = 5) and +Fe (N = 5) treatment tanks during the 14-day experimental period (mean ± SD). The *p*-value on each panel indicates the significance level (*, α = 0.05) of the treatment effect based on repeated measures ANOVA.

Copepod nauplii started to appear 4 days after fertilization, peaked after 8 days, and declined thereafter under both treatments (Figure 4A), with no significant difference in abundance between the control and +Fe tanks (*p* > 0.05). Adult copepod abundance (including copepodids) was significantly higher in the +Fe treatment tanks, especially after day 8, and remained so until the end of the experiment (Figure 4B). Total copepod dry weight (Figure 4C) followed the same trend as for adults and copepodids and was significantly higher in the +Fe treatment tanks than in the controls (*p* < 0.05).

Non-copepod zooplankton, mainly ciliates: *tintinnids* and *Strombidium* spp., appeared early during the experiment but decreased as copepods became dominant (Figure 4D). Their density fluctuated and was significantly higher in +Fe treatment tanks than in the controls (*p* < 0.05).

**Figure 5:**
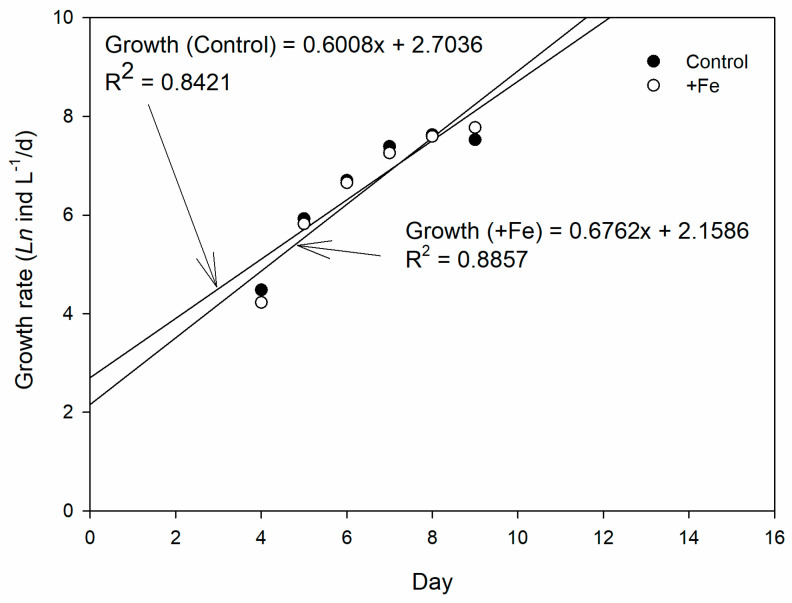
The growth rate of copepod in the control (N = 5) and +Fe (N = 5) treatment tanks during the 14-day experimental period.

The population growth rates from the exponential phase of population growth (Day 4–9) were 0.6008x + 2.7036 and 0.6762x + 2.1586, for control and +Fe treatment, respectively (Figure 5).

The calculation of total costs and net profits showed that the cost of producing copepods was 23% higher with the +Fe treatment (NT$0.69 ± 0.10 g^−1^ dry weight) than in the controls (NT$0.56 ± 0.07 g^−1^ dry weight), but the expected net profit was also significantly (97%) higher with the +Fe treatment (NT$11.04 ± 2.49 g^−1^ dry weight) than for the controls (NT$5.61 ± 0.83 g^−1^ dry weight) (*t*-test, *p* < 0.05).

## 4. Discussion

Copepods are a proven live feed for newly hatched fish larvae, especially for some larvae of coral reef fish with small mouth gapes [9,37,64,65,66]. They can enhance the survival and growth rate of fish larvae and reduce the incidence of deformities. Access to a reliable supply of copepods on a commercial scale has been a bottleneck faced by aquaculturists hoping to use copepods in their operations on a daily basis. To overcome this shortfall, several methods, as well as specialized equipment for the large-scale production of copepods, have been devised [18,25,67,68]. For example, Prado-Cabrero, Herena-Garcia, and Nolan [68] designed a monoalgal bioreactor that uses the microalga *Tetraselmis chui* to continuously produce the harpacticoid copepod *Tigriopus californicus* [68]. Sarkisian, Lemus, Apeitos, Blaylock, and Saillant [67] developed an indoor batch-culture system for intensive production of the calanoid copepod *A. tonsa*, featuring integrated grow-out and egg-production units that can produce nauplii daily. Hong and Tew [25] did a pilot study adding a precise ratio of inorganic nitrogen and phosphorus fertilizers to culture tanks in order to mass-produce the calanoid copepod *P. annandalei*. All of these efforts were aimed at helping to regularize the mass production of copepods to supply the aquafeed industry with marine larval fish culture.

Our previous study [25] showed that inorganic fertilization of culture tanks could produce pathogen-free copepods at a cheaper cost per unit output than the commonly used organic fertilization method. However, the Chl a concentration of phytoplankton usually declined rapidly in the tanks even when nitrogen and phosphorus were still abundant [4,25], thus affecting copepod production. Since iron is involved in phytoplankton photosynthesis [45], and is a trace element necessary for phytoplankton growth [49,50,51], we modified our inorganic fertilization method by adding iron into the copepod culture tanks. The concentration we employed (10 μg L^−1^ Fe) was higher than that employed by previous researchers: 0.16 μg L^−1^ Fe by Watson et al. [54] to stimulate limnetic phytoplankton, 0.06 μg L^−1^ Fe by Takeda [48] to raise oceanic diatoms, and 5.60 μg L^−1^ Fe by Chen et al. [55] to enhance marine phytoplankton. Since we cannot be certain of the concentration of Fe that will adequately enhance phytoplankton productivity, we chose a much higher but economically feasible concentration. Different iron concentrations should be tried in future studies to lower the cost of copepod production. 

In this study, instead of inoculating mono-species culture, the random microalgae from natural seawater were induced by inorganic fertilizer. Since the microalgae composition in the coastal water is commonly seasonal- and regional-specific, one might suspect that the outcomes from using the inorganic fertilization method will vary at different places and seasons, making it unreliable in producing copepods. To the contrary, several larviculture experiments using this method have shown that the phytoplankton and zooplankton productions were consistent and reliable, and higher survival rates of coral reef fish larvae were achieved, irrespective of when and where it was applied [4,33,37,69]. Earlier studies showed unicellular diatoms such as *Coscinodiscus* spp., *Navicula* spp., and *Nitzschia* spp. out-competed filamentous diatom *Chaetoceros* spp. under inorganic fertilization treatment [4,33], whereas in the present study, the unicellular green algae *Chlamydomonas* spp. succeeded *Chaetoceros* spp. The taxonomical compositions of algae were different during different experiments, but it was always smaller unicellular phytoplankton that prevailed. By controlling the ratio and concentration of nitrogen to phosphorus in the water column, we would expect to have phytoplankton compositions of desired sizes, albeit different species in different places or seasons. This method will liberate aquaculturists worldwide from having to monoculture certain algal species in order to feed their monoculture zooplankton, which is then fed to the fish larvae.

During the present experiment, the tanks were set up outdoors and were thus subject to various environmental stresses. As a result of exposure to direct sunlight, for example, the water temperature at 1100~1300 h could reach 38 °C. Fitness trade-offs have been reported for the intertidal copepod *T. californicus* under extremely high temperatures [70], but for other copepod species such conditions can be *ameliorated by providing sufficient* food, similar to what was found by Hammock, et al. [71] in that low food availability narrows the tolerance of the copepod *Eurytemora affinis* to salinity [71]. The high Chl a concentration in our study indicates that there was a high abundance of phytoplankton serving as food for the copepods, which likely offset any temperature stress experienced by the copepods in this study. Moreover, as the temperature goes up, dissolved oxygen tends to be reduced to the detriment of many aquatic organisms [72]. In our study, the mean dissolved oxygen was always 5.00 mg L^−1^ or higher, thus, above the level at which low oxygen negatively affects the survival and egg production of copepods [73]. This was also likely due to the presence of a high density of phytoplankton in the culture tanks and the resulting production of oxygen as a product of photosynthesis. A high pH can result in a cessation of egg production or an increased nauplii mortality in some species of copepod [74], and our previous study of organic fertilization of culture tanks showed that the growth of nauplii into copepodites and adult copepods was constrained at pH 9.5 [25]. The latter effect was, however, negligible in the present study when iron was added along with inorganic nitrogen and phosphorus. Since nutrient deficiency may reduce the efficiency of energy transfer at the base of food webs by altering the chemical composition of phytoplankton [75], the high quantity of the phytoplankton in our study may have alleviated the otherwise negative impact of environmental stresses on the copepod population.

The concentrations of nutrients such as NO_3_-N and PO_4_-P were significantly lower overall in the +Fe treatment tanks than in the control tanks, with the beginning of their decline co-occurring with or immediately preceding the peaks on day 9 in Chl a concentration attributable to smaller-sized algae and in copepod density. Based on the results, we would suggest applying higher concentrations of nitrogen and phosphorus in the future study along with the addition of iron to further enhance the phytoplankton growth. Although excess phosphate could enhance the massive growth of benthic filamentous algae, such as *Tribonema* spp. [25,76,77], and suppress the growth of phytoplankton which adversely affect the growth of copepod larvae and adults, however, with or without the addition of iron, we observed no significant growth of filamentous algae during the present study, but only unicellular algae. This further strengthens our argument that precise adjustment of the nutrient concentrations and N:P ratio, as enabled by the inorganic fertilization method, provides better conditions for the growth of copepod nauplii than organic fertilization does.

## 5. Conclusions

Commercially available copepod products are produced by applying various organic materials as fertilizer to extensive outdoor culture ponds or as a by-product of fish ponds. As a result, the quantity and quality of the products are inconsistent. Our previous study found that using inorganic fertilizer was better as compared to the organic method in terms of quantity and quality of copepod production. In this study, we further found that by adding iron as part of an inorganic fertilization protocol for rearing copepods in large culture tanks, the growth of phytoplankton lasted much longer, and a significantly higher density of copepodids and adult copepods could be obtained. Adding iron increased the cost, but the net expected profit was almost twice as high with the addition of iron than without. We contend that inorganic fertilization with the addition of iron, if conducted at a large enough scale, could be an effective method for the mass production of copepods as live feed for use in fish larviculture. 

## Data Availability

Data will be provided whenever requested.
